# Superparamagnetic Iron-Oxide Nanoparticles Synthesized via Green Chemistry for the Potential Treatment of Breast Cancer

**DOI:** 10.3390/molecules28052343

**Published:** 2023-03-03

**Authors:** Neha Tyagi, Priya Gupta, Zafar Khan, Yub Raj Neupane, Bharti Mangla, Nikita Mehra, Tanya Ralli, Abdulsalam Alhalmi, Asgar Ali, Omkulthom Al Kamaly, Asmaa Saleh, Fahd A. Nasr, Kanchan Kohli

**Affiliations:** 1 Department of Pharmaceutics, School of Pharmaceutical Education & Research, Jamia Hamdard, New Delhi 110062, India; 2 Department of Pharmaceutical Sciences and Experimental Therapeutics, College of Pharmacy, The University of Iowa, Iowa City, IA 52242, USA; 3 Department of Pharmaceutics, Delhi Pharmaceutical Sciences and Research University (DPSRU), New Delhi 110017, India; 4 Innovation and Science, Amway Global Services India, Gurugram 122001, India; 5 Department of Pharmaceutical Sciences, College of Pharmacy, Princess Nourah Bint Abdulrahman University, Riyadh 11671, Saudi Arabia; 6 Department of Pharmacognosy, College of Pharmacy King Saud University, Riyadh 11451, Saudi Arabia; 7 Department of Pharmaceutics, Lloyd Institute of Management and Technology (Pharm.), Greater Noida 201306, India

**Keywords:** SPIONs, green chemistry, superparamagnetic iron-oxide nanoparticles, serum albumin, breast cancer, tamoxifen

## Abstract

In the emerging field of nanomedicine, nanoparticles have been widely considered as drug carriers and are now used in various clinically approved products. Therefore, in this study, we synthesized superparamagnetic iron-oxide nanoparticles (SPIONs) via green chemistry, and the SPIONs were further coated with tamoxifen-conjugated bovine serum albumin (BSA-SPIONs-TMX). The BSA-SPIONs-TMX were within the nanometric hydrodynamic size (117 ± 4 nm), with a small poly dispersity index (0.28 ± 0.02) and zeta potential of −30.2 ± 0.09 mV. FTIR, DSC, X-RD, and elemental analysis confirmed that BSA-SPIONs-TMX were successfully prepared. The saturation magnetization (M_s_) of BSA-SPIONs-TMX was found to be ~8.31 emu/g, indicating that BSA-SPIONs-TMX possess superparamagnetic properties for theragnostic applications. In addition, BSA-SPIONs-TMX were efficiently internalized into breast cancer cell lines (MCF-7 and T47D) and were effective in reducing cell proliferation of breast cancer cells, with IC_50_ values of 4.97 ± 0.42 μM and 6.29 ± 0.21 μM in MCF-7 and T47D cells, respectively. Furthermore, an acute toxicity study on rats confirmed that these BSA-SPIONs-TMX are safe for use in drug delivery systems. In conclusion, green synthesized superparamagnetic iron-oxide nanoparticles have the potential to be used as drug delivery carriers and may also have diagnostic applications.

## 1. Introduction

Breast cancer (BC) is the most commonly occurring type of cancer and the second most common cause of cancer-related deaths, with 1 million new cases and over 68,500 deaths globally each year [1,2]. Approximately 3.2 million people are anticipated to be affected with female breast cancer worldwide by 2050 [3]. Furthermore, this heterogeneous disease has surpassed lung cancer incidence. The commonly used conventional treatment strategies employed in modern medicine for the treatment of BC include chemotherapy, radiation, endocrine therapy, surgery, and hormone therapy [4,5]. Hormonal therapy is the most common treatment option due to the increased cases of estrogen/progesterone-positive type BC, which now accounts for approximately 70% of cases [6,7,8]. Of the treatment approaches available for the management of estrogen-positive BC, chemotherapy with tamoxifen (TMX), a selective estrogen receptor modulator (SERM); fulvestrant, a selective estrogen receptor down-regulator; and letrozole, an aromatase inhibitor, represent some of the most frequently used treatment strategies [9].

Since the approval of TMX in 1988 by the US FDA, it has been widely used as a chemotherapeutic agent for the management of breast cancer [10], and is responsible for the reduction in the annual rate of mortality by nearly 51%. It is now considered the “gold standard therapy” in both pre- and post-menopausal women for the effective treatment of estrogen-positive BC [4], and its mechanism of action includes attachment to estrogen receptors, thereby instigating a conformational change and finally inducing a change in the expression of the genes associated with estrogen [11]. Despite its advantages in therapeutic use, TMX belongs to BCS class II [12]; it still has restricted therapeutic applications due to low solubility and low oral bioavailability (<20%) [13], affecting its efficacy and tolerance as it undergoes first-pass metabolism after oral administration and resulting in substantial drug consumption [14]. Additionally, TMX accumulates in the body, thereby elevating the risk of endometrial cancer, even though the effect is dose- and duration-dependent [15]. In addition, there are significant challenges associated with the use of conventional cancer treatment strategies, such as refractoriness and systemic toxicities. These challenges have paved the way for nanotechnology to alleviate the severity of disease and improve its diagnosis. Further, with the development of nanodrug delivery, accurate targeting of the malignant cells with minimal side effects can be achieved, which can be attributed to the “ease of crossing” exhibited by nanomaterials due to their nanosize [16,17].

Though a plethora of chemotherapeutic agents are being used for the treatment of cancer, due to their adverse side effects combined with their non-specific targeting that also affects healthy cells, the sensitivity of the drug often results in insufficient outcomes [18]. In this context, typical nanodrug delivery systems, namely lipid nanoparticles, liposomes, dendrimers, polymeric nanoparticles, inorganic nanoparticles, and polymer–drug conjugates, have been thoroughly investigated [19,20,21,22,23].

Superparamagnetic iron-oxide nanoparticles (SPIONs) are one such nanocarrier system that has been recently gaining the attention of researchers worldwide due to its multifaceted potential in hyperthermia, gene delivery, imaging, and drug delivery [24,25]. Additionally, in the field of nanomedicine, SPIONs are used for magnetic cell sorting, separation of bioactive molecules from blood and tissue homogenates, and as contrast materials in magnetic resonance imaging (MRI) [26,27]. Owing to their exceptional superparamagnetic property, which aids in their accumulation inside a particular biological tissue and helps with their tracing under the influence of an external magnetic field, they have become the most extensively studied nanocarrier [28,29,30]. Furthermore, given their distinct magnetic characteristics and their functionalization with various polymers, SPIONs can be used as an efficient drug delivery system and in tumor therapy, wherein they are highly regarded by researchers for potentiating the delivery of various chemotherapeutics [31], representing a new paradigm of possibilities in the treatment of cancer [32]. Moreover, given the vast surface of the SPIONs, they are capable of substantially increasing the possibility of the covalent attachment of several kinds of receptors, ligands, peptides, or antibodies, which further enables their specific receptor binding and controlled release of the drug, a phenomenon termed “controlled drug release”. Compared to conventional chemotherapy, this aids in overcoming non-specific targeting, as well reducing the need for relatively larger treatment doses, since drug-loaded SPIONs exhibit specific targeting capacity to particular cells, tissues, or organs [33,34,35]

SPIONs tend to aggregate without an external coating on their surface and are lipophilic, and upon their entrance into the blood, they eventually become coated by plasma proteins, resulting in opsonization that can be minimized or even prevented by employing a hydrophilic coating that is capable of decreasing the aggregation of SPIONs by interacting with the electrostatic charges or by causing steric hindrances [36]. Thus, despite their unique theranostic characteristics, SPIONs’ low biocompatibility and tendency to aggregate often limit their utility in biomedical applications [37]. This points to the importance of successfully engineering the surface of these nanocarriers [38,39]. SPIONs’ surfaces are generally coated using biocompatible polymers, including polyethylene glycol (PEG), bovine serum albumin (BSA), and chitosan, either by physical or chemical coating, for biomedical purposes to alleviate these limitations [26]. The surface modification of SPIONs can be performed by coating them with non-toxic and biocompatible polymers that direct the nanoparticles to the target site of the disease [40]. The decoration of these SPION nanoparticles with bioactive chemicals leads to target site selectivity and prevents the interaction of healthy cells with tissue and drug carriers, achieved by appropriate surface coatings of biocompatible proteins such as albumins. Moreover, albumin, due to its nature to interact with biological receptors, can act as carrier to deliver nanoparticles to the target site, and surface modification of nanoparticles with albumin will help to accumulate nanoparticles at the target site [41,42]. These systems have great potential as they last longer in blood circulation and have better stealth properties when compared to non-coated nanoparticles. In addition, the protein coating can also be used as a theranostic vehicle for drugs and other targeting agents [43].

Based on this, our research focused on developing BSA-coated SPIONs (BSA-SPIONs) as an effective drug nanocarriers to deliver drugs to the target site [44,45]. The colloidal stability of the nanoparticle was maintained by the BSA coating, which also functioned as a standard additive for the cell culture experiment serum. BSA has a similar chemical composition to HSA and is preferred because of its cost-effectiveness; additionally, it is non-toxic and non-immunogenic [46]. A study by Yamasaki et al. reported that HSA enhanced the dissolution and solubility properties of praziquantel, leading to enhanced bioavailability [47]. Albumin-coated nanoparticles offer various advantages due to the different sites for binding, making them a potential carrier system. In another study conducted by Yu, Gonzalez-Moragas et al., the partial digestion of uncoated SPIONs in an acidic gastric environment was compared to BSA-coated SPIONs, with favorable results [48].

Based on these fundamental concepts, we developed a well-defined BSA corona on the surface of SPIONs, designed to enhance the stability of SPIONs in which TMX was conjugated (BSA-SPIONs-TMX). Green chemistry was utilized in the fabrication of the SPIONs, and the fabricated nanoparticles were characterized for various parameters, including particle size, zeta potential, surface morphology, solid-state characterization, elemental analysis, magnetic properties, and in vitro release. Further, a cellular uptake study and cell cytotoxicity study were performed in breast cancer cell lines (MCF-7 and T47D cells). An acute toxicity study on rats was also performed.

## 2. Results and Discussion 

### 2.1. Characterizations of Green Synthesized SPIONs

**Figure 1:**
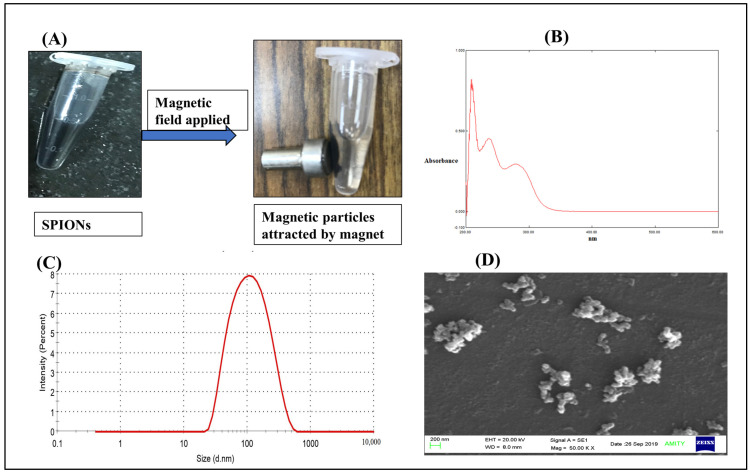
Characterizations of green synthesized SPIONs. (**A**) Green synthesized SPIONS using (-) EGCG showed magnetic properties in the presence of an externally applied magnetic field. (**B**) UV-visible absorption spectra of SPIONs in the UV-visible range (200–600 nm) showed maximum absorption (characteristic) peaks at 210, 236, and 278.5 nm. (**C**) Particle size distribution (hydrodynamic diameter) profile of SPIONs. (**D**) SEM image of SPIONs (scale bar 200 nm).

SPIONs synthesized by the co-precipitation method via a controlled oxidation reduction of the iron salt precursor using (−) EGCG were attracted towards an externally applied magnetic field, as shown in Figure 1A, suggesting that SPIONs possess the excellent magnetic properties necessary for biomedical applications, such as targeted drug delivery [49] As shown in Figure 1B, the UV-visible spectra of SPIONs showed absorption at 210, 236, and 278.5 nm, which are the characteristic absorption peaks of iron-oxide nanoparticles, indicating that iron-oxide nanoparticles (SPIONs) were formed [49,50,51,52]. Furthermore, we characterized the SPIONs in terms of size (hydrodynamic diameter) and surface charge and found them to be ~90 nm [53] with a PDI of 0.27 (Figure 1C) and surface charge of –13.6 mV. These SPIONs were in the nanometric size range with good PDI, indicating they were monodispersed in nature [27]. However, SEM images showed some sort of aggregation (Figure 1D), which corroborated with the literature suggesting that these SPIONs tend to aggregate and need a coating to prevent this. This agglomeration of magnetic particles could be due to the presence of high surface energy between the prepared magnetic nanoparticles and the presence of magnetic dipole–dipole interactions [54]. Therefore, in the next step, we coated the SPIONs with the BSA protein, with the aim of preventing aggregation during storage.

### 2.2. Characterizations of Green Synthesized TMX-Conjugated BSA-Coated SPIONs

**Figure 2:**
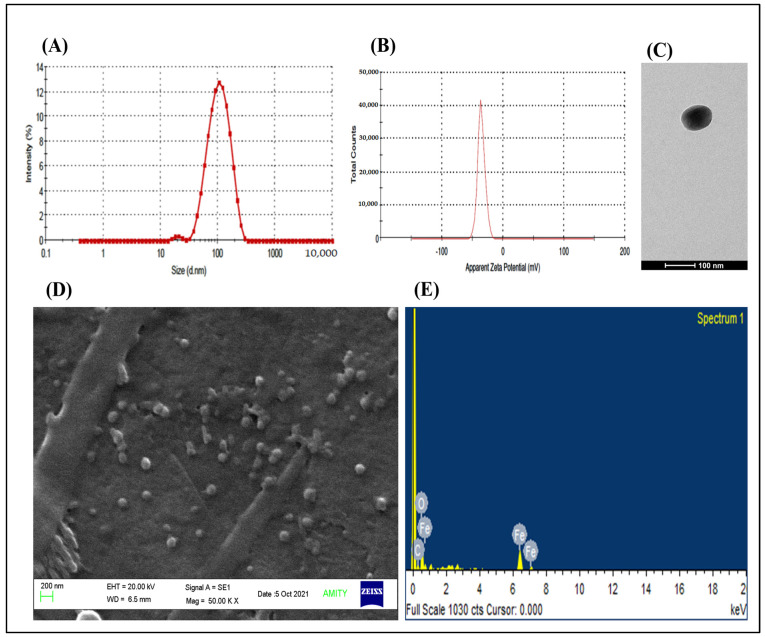
Characterizations of green synthesized TMX-conjugated BSA-coated SPIONs. (**A**) Particle size (hydrodynamic diameter) distribution profile of BSA-SPIONs-TMX. (**B**) Zeta potential distribution profile of BSA-SPIONs-TMX. (**C**) TEM image of BSA-SPIONs-TMX (scale bar 100 nm). (**D**) SEM image of BSA-SPIONs-TMX (scale bar 200 nm). (**E**) EDX analysis of BSA-SPIONs-TMX using SEM-EDX.

The particle size (hydrodynamic diameter) and PDI of TMX-conjugated BSA-coated SPIONs (BSA-SPIONs-TMX) was observed to be 117 ± 4 nm and 0.28 ± 0.02 (Figure 2A), respectively, and the surface charge of BSA-SPIONs-TMX was found to be –30.2 ± 0.09 mV (Figure 2B). There was a slight increment in the particle size of the BSA-SPIONs-TMX compared to SPIONs, indicating that the coating was successfully applied to the SPIONs, which was further confirmed with a decrease in the surface charge of the BSA-SPIONs-TMX. The more negative surface charge observed in the BSA-SPIONs-TMX could be because of the presence of the carboxylic acid on the surface of BSA, which was used for the stabilization of the SPIONs [55]. As confirmed with TEM imaging (Figure 2C), BSA-SPIONs-TMX were also in the nanometric size range and were spherical in nature. Moreover, SEM imaging (Figure 2D) confirmed that the BSA coating prevented the aggregation of the SPIONs. The sol-gel, chemical reduction, chemical coprecipitation, hydrothermal, flow injection, sonochemical decomposition, electrochemical, oxidation, aerosol/vapor phase, nanoreactor, and simple fluid methods are various methods that have been reported for the synthesis of iron-oxide nanoparticles [50]. The green synthesis method has gained popularity as it utilizes plant extract, which eliminates the use of harmful reducing agents [51]. The plant extract contains a variety of phytochemicals such as polyphenols, flavonoids, terpenoids, and phenolic acids, which act as reducing and capping agents. For instance, iron-oxide nanoparticles were synthesized from ferric chloride hexahydrate using papaya leaf extract as the reducing agent [52].

Next, we performed an elemental analysis of the BSA-SPIONs-TMX via SEM-EDX analysis, where the elemental composition of the iron (Fe) and oxygen (O) in the iron-oxide nanoparticles (BSA-SPIONs-TMX) was quantified [56]. As shown in Figure 2E, the percentage of Fe and O in the BSA-SPIONs-TMX was found to be 35.86% and 41.33%, respectively, due to the iron oxide present in the SPIONs. In the previous study, ~32.7% of iron and ~31.03% of O were found in the iron-oxide nanoparticles prepared via the green synthesis method [57]. We observed ~22% carbon (C) in the BSA-SPIONs-TMX, which could have been due to the presence of an albumin coating on the BSA-SPIONs-TMX, and this was similar to previous reports, as carbon is one of the components of proteins in the albumin used for the coating [58]. 

Then, we quantified the amount of TMX entrapped in the BSA-SPIONs-TMX at ~79.6%, suggesting that a good amount of TMX was entrapped in the BSA-SPIONs-TMX. Overall, our data suggested that SPIONs coated with BSA were within the nanometric range and were more stable than SPIONs.

### 2.3. Magnetic Measurement

**Figure 3:**
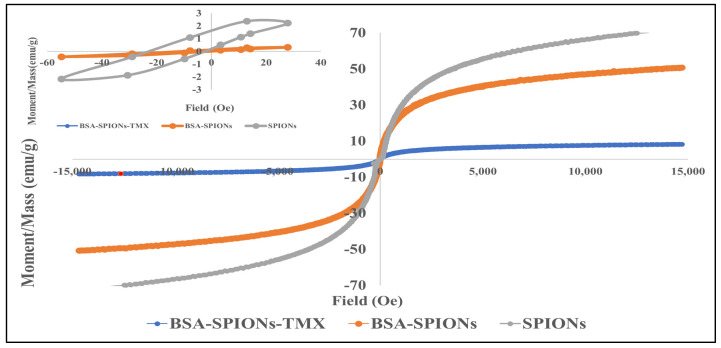
Field dependence of magnetization green synthesized SPIONs, BSA-SPIONs, and BSA-SPIONs-TMX measured in the applied magnetic field (Oe) at room temperature.

Figure 3 shows the room temperature magnetic hysteresis loop of SPIONs, BSA-SPIONs, and BSA-SPIONs-TMX, which demonstrates the superparamagnetic behavior of green synthesized magnetic nanoparticles (SPIONs). The hysteresis loop was zoomed in around zero field to study the magnetic properties (inset Figure 3). The saturation magnetization (Ms) of SPIONs was found to be 72.05 emu/g, whereas the Ms of BSA-SPIONs was found to be 50.70 emu/g, which was decreased as compared to SPIONs due to coating with BSA. The Ms observed for BSA-SPIONs-TMX was 8.31 emu/g. The reduction in Ms after coating could be due to some dead layer generated after coating in the magnetic nanoparticles [59,60]. Moreover, the Ms value reported in the literature for coated magnetic nanoparticles was in the range of 2.5 to 57 emu/g [61]. At room temperature, these BSA-SPIONs-TMX are superparamagnetic, making them beneficial for targeted drug delivery, and there are negligible chances of the agglomeration of particles because of the magnetic dipole attraction. These nanoparticles almost do not possess residual magnetization unless an external magnetic field is applied to them. 

### 2.4. FTIR Spectral Analysis

**Figure 4:**
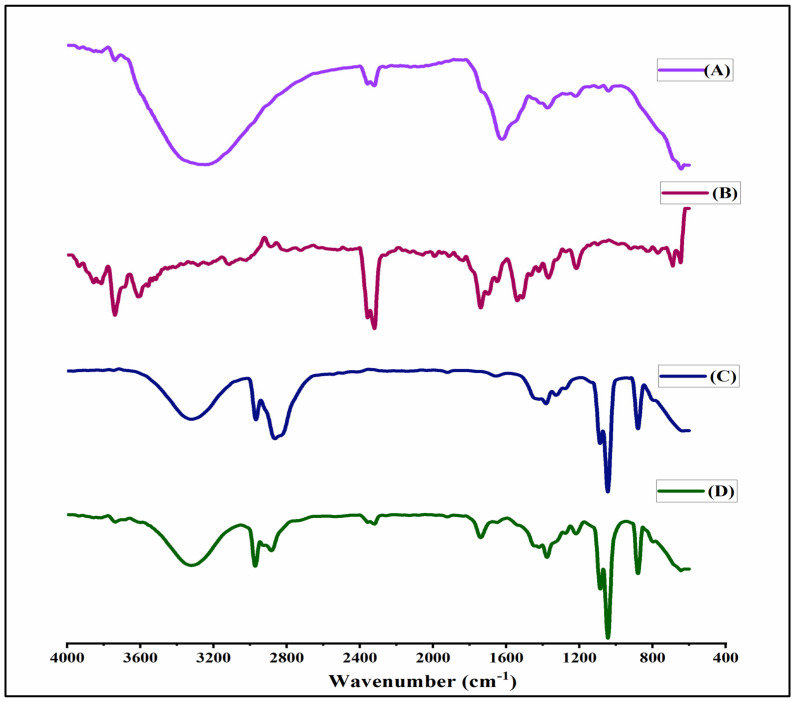
FTIR spectra of (**A**) SPIONs (10 mg), (**B**) TMX (10 mg), (**C**) BSA (10 mg), and (**D**) BSA-SPIONs-TMX (10 mg).

The FTIR spectra of SPIONs, BSA, TMX, and BSA-SPIONs-TMX are depicted in Figure 4. The FTIR spectra of SPIONs showed various characteristics peaks at different regions: 3550–3350 cm^−1^ related to the O–H stretching vibrations (polyphenolic group), 2358–2320 assigned to uncorrected carbon dioxide, a peak at 1373 cm^−1^ attributed to the C–H rocking vibration of methyl groups, and a peak at 800–400 cm^−1^ zone characteristic of the Fe–O bond, with the strongest peak at 638 cm^−1^ [61]. The FTIR spectra of SPIONs indicated the existence of polyphenols, flavonoids, and other biomolecules, and the formation of SPIONs during the oxidation reduction process. The function of polyphenols in SPIONs is to function as a capping and reducing agent. The FTIR spectra of TMX showed characteristic peaks at 1739 cm^−1^ due to the C=O group, N-H bending at 1539 cm^−1^, phenyl ring substitution at 768–700 cm^−1^, and C-N stretch at 1216 cm^−1^. Similarly, in the FTIR spectra of BSA, the bands were found at 3322 cm^−1^, 2968 cm^−1^, 1656 cm^−1^, 1040 cm^−1^, and 880 cm^−1^, corresponding to amide A, amide B, C=O stretching, amide III, and C=C, respectively. The bands at 1656 cm^−1^ were assigned as asymmetric, and 1382 and 1327 cm^−1^ corresponded to symmetric stretches of the carboxylate group of BSA [62]. The FTIR spectra of BSA-SPIONs-TMX showed band at 3300 cm^−1^ due to BSA (N-H). Our results suggested that BSA can function as carrier macromolecule for TMX and protects SPIONs against aggregation, as well as enhancing the stability of metal oxide nanoparticles. 

### 2.5. Differential Scanning Calorimetry

**Figure 5:**
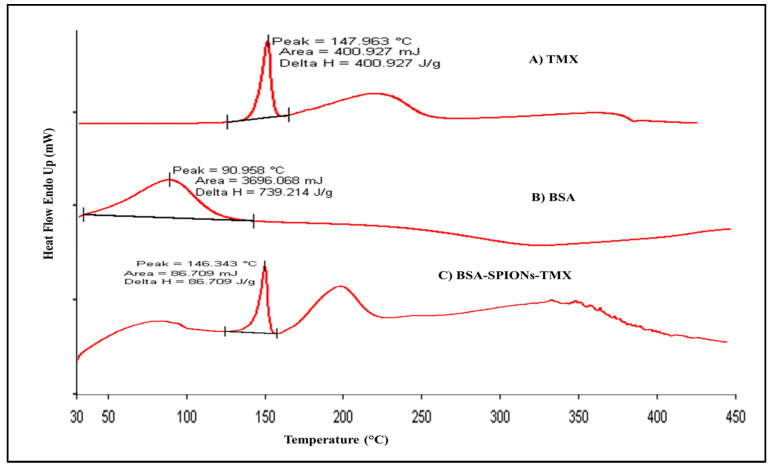
DSC thermogram of TMX, BSA, and BSA-SPIONs-TMX.

The DSC study demonstrated the thermo-analytical behavior of TMX, BSA, and BSA-SPIONs-TMX, as depicted in Figure 5. It also indicated whether any modification in the physical properties of drugs and excipients occurred during the formulation process. The BSA peak is much broader could be due to entrapment of TMX within the BSA-SPIONs matrix, and the peak around 200 °C could be due to the presence of excipients. The thermogram of TMX and BSA exhibited a sharp endothermic peak at 147.9 °C and a broad peak at 90.9 °C, corresponding to their melting points. In the final formulation (BSA-SPIONs-TMX), the DSC thermogram showed a TMX peak at 146.3 °C and broad peak of BSA, suggesting that formulation was stable during the production process, and both TMX and BSA were well retained in the final formulation.

### 2.6. X-ray Diffraction

**Figure 6:**
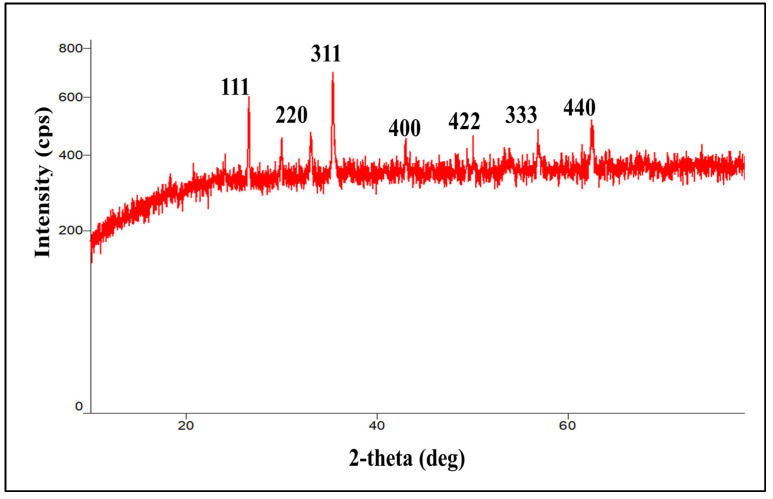
X-ray diffractogram of SPIONs.

The crystalline nature of SPIONs was characterized via XRD. As shown in Figure 6, the XRD patterns of SPIONs display several relatively strong reflection peaks in the 2theta region of 5–60°. All of the (111), (220), (311), (400), (422), (333), and (440) reflection peaks were found to be well indexed to the inverse cubic spinel structure of Fe_3_O_4_ (JCPDS cardno.75-1610) based on peak positions and relative intensities, proving that the nanoparticles synthesized here are indeed Fe_3_O_4_ nanoparticles [63]. Such reflection peaks were also reported in previous study, where Fe_3_O_4_ spherical magnetic nanoparticles were synthesized using Syzygium cumini seed extract [61]. A single strong peak corresponding to magnetite was also reported at 2theta = 35.28° in magnetite nanoparticles coated with dextran in reference to ICCD 01-073-9877 [64]. Once again, this confirmed that iron-oxide magnetic nanoparticles can be synthesized by this facile and green method. 

### 2.7. Long-Term Stability Study

The long-term stability of BSA-SPIONs-TMX was assessed over three months under refrigeration (4 °C) and at room temperature (25 °C/60% relative humidity). At predetermined time intervals, samples were withdrawn and analyzed for particle size and zeta potential. As shown in Table 1, the particle size and zeta potential of BSA-SPIONs-TMX after 3 months were within the acceptable range (below 200 nm for nanoparticles), suggesting that these SPIONs can be stored for a long period of time and used when required. The samples were reconstituted in Milli-Q water for particle size and zeta potential determination. 

### 2.8. In Vitro Drug Release

**Figure 7:**
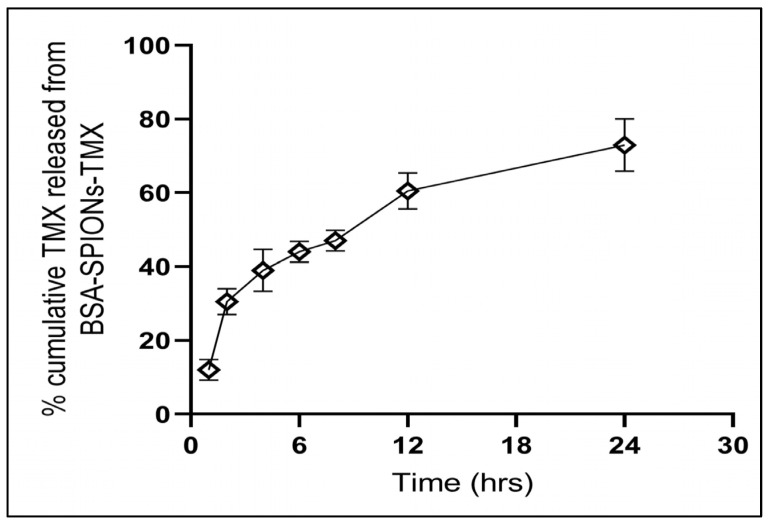
Percentage cumulative TMX released from BSA-SPIONs-TMX within 24 h. Data represent mean ± SD (*n* = 3).

The in vitro release kinetic pattern of TMX from BSA-SPIONs-TMX was assessed in phosphate buffer solution (pH 7.4) using the dialysis method, in which ~8–10% of TMX was released from BSA-SPIONs-TMX within the first 2 h (Figure 7). After 24 h, ~74–76% of the TMX was released from the BSA-SPIONs-TMX, suggesting a sustained drug delivery pattern of BSA-SPIONs. Such a sustained drug delivery system is particularly important for targeted nano-drug delivery where the drug has to reach a target site.

### 2.9. Intracellular Uptake Study

**Figure 8:**
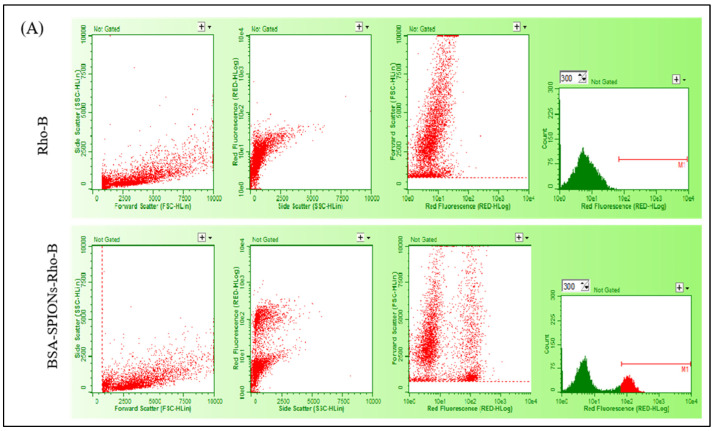

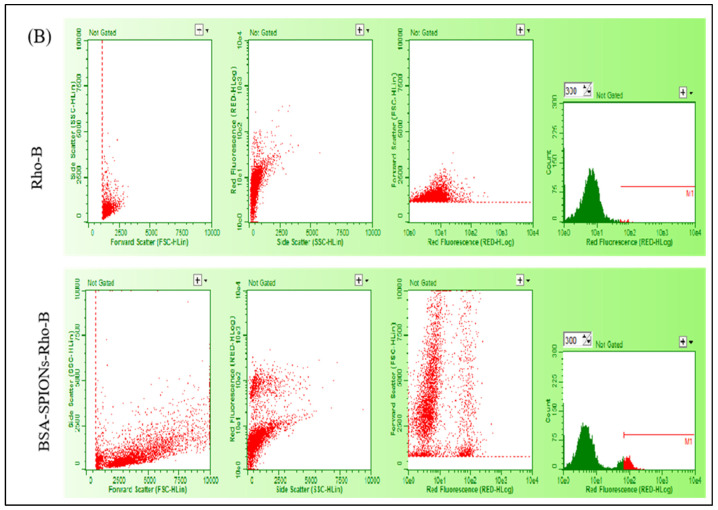
Cellular uptake profiles of Rho-B and BSA-SPIONs-Rho-B from different cells: (**A**) MCF-7 cells, and (**B**) T47D cells, showing side scatter v/s forward scatter, fluorescence v/s side scatter, forward scatter v/s fluorescence, and fluorescence histogram (log).

MCF-7 and T47D cells were incubated with Rhodamine-B-loaded BSA-SPIONs, and the intracellular uptake of BSA-SPIONs was determined using flow cytometry after 4 h of incubation. BSA-SPIONs must be able to be internalized into various types of cells in the maximum amount and as quickly as possible to be an ideal drug delivery system. SPIONs were reported to internalize into cells via endocytosis more efficiently in cancer cells compared to normal cells, as evidenced by the qualitative, semi-quantitative, and quantitative analysis [65,66]. In our study, the internalization of BSA-SPIONs was evident from the side scatter plot of the fluorescence intensity in MCF-7 and T47D cells, as shown in Figure 8, which was higher compared to plain Rho-B. The ROD-B dye has an emission and excitation spectrum. Interaction between a laser beam and the fluorescing cells or particles causes a pulse of photons to occur over time (peak). They are detected by PMTs as a voltage pulse in the form of an event. The intensity of the fluorescence is directly related to the voltage pulse area. The M1 events provide an indication of number of particles internalized in the cells and is correlated by higher fluorescence intensity. The number of cells stain due to dye causes the curve plot to shift to right, which is determined by an increase in number of M1 events, which is directly proportional to the cellular uptake. The number of events attributed increases with fluorescence. 

The M1 events for the Rho-B solution and BSA-SPIONs were found to be 5 and 1230, respectively, for MCF-7 cells, and 15 and 566, respectively, for T47D cells, suggesting a significantly higher uptake of BSA-SPIONs compared with the Rho-B solution in both MCF-7 and T47D cells. BSA-SPIONs showed better internalization as compared to plain rhodamine-B solution due to the nanosize of the formulation, and because the BSA coating in the BSA-SPIONs facilitated better adherence to the cell membrane. This indicated that BSA-SPIONs could be an effective drug carrier for the treatment of cancer. 

### 2.10. Cell Cytotoxicity Assay

**Figure 9:**
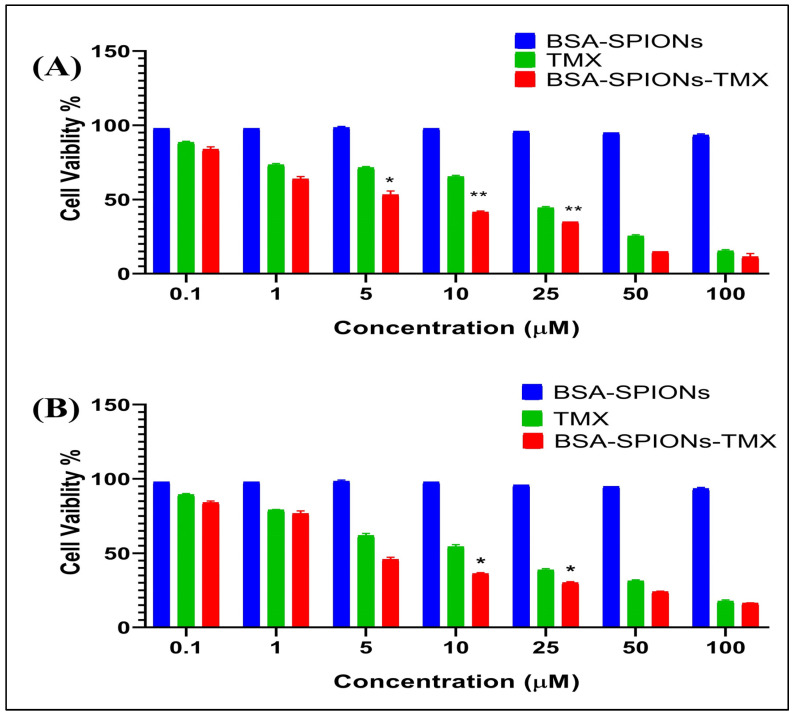
Percentage cell viability profile of TMX and BSA-SPIONs-TMX against (**A**) MCF-7 cell lines and (**B**) T47D cell lines (mean ± SD, *n* = 3); Note: (* indicates *p* < 0.05 compared with TMX and ** indicates *p* < 0.01) compared with BSA-SPIONs-TMX.

The cell cytotoxicity (MTT) assay was performed to examine the in vitro anti-tumor activity of the TMX suspension and BSA-SPIONs-TMX in human breast cancer cell lines (MCF-7 and T47D cells). As shown in Figure 9, BSA-SPIONs-TMX showed comparatively better cytotoxic activity in both cell lines than the TMX suspension. The IC_50_ value of the TMX suspension and BSA-SPIONs-TMX was determined to be 15.12 ± 0.110 μM and 4.97 ± 0.417 μM, respectively, for MCF-7 cell lines, whereas the IC_50_ value of TMX suspension and BSA-SPIONs-TMX was found to be 15.30 ± 0.310 μM and 6.29 ± 0.213 μM, respectively, for T47D cell lines. This could be due to higher internalization of BSA-SPIONs-TMX into the cells (corroborated with our intracellular uptake study) compared to the TMX suspension, which enhanced the anti-proliferation action of TMX. Simultaneously, we also assessed the cell cytotoxicity activity of BSA-SPIONs, and interestingly, we did not observe any cell cytotoxicity from BSA-SPIONs (Figure 9), suggesting that they are safe for theranostic applications. The IC50 was determined using Graph-Pad software and comparing was done between two samples through the Student’s *t*-test having a *p* value of <0.05 and 0.01.

### 2.11. Acute Toxicity Study

**Figure 10:**
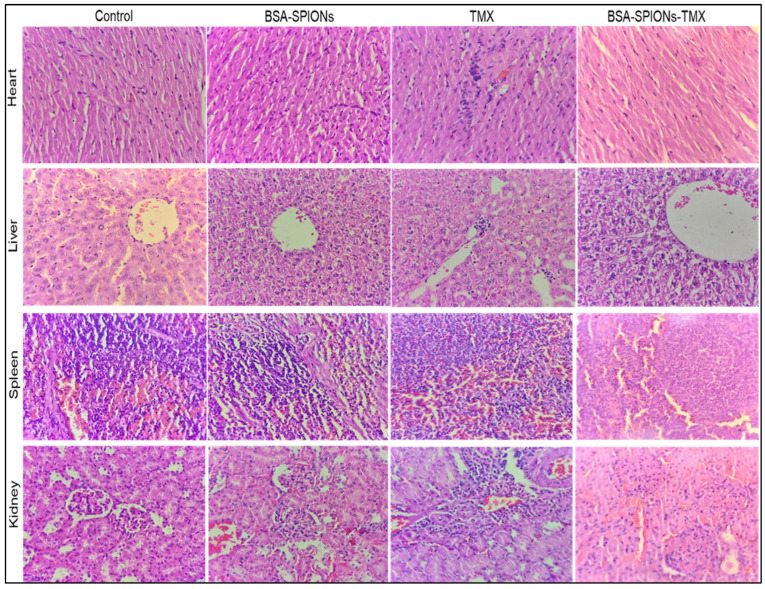
Representative photomicrographs of histopathology (40×) of a cross-section of the heart, liver, spleen, and kidney from the control, BSA-SPIONs-, TMX-, and BSA-SPION-TMX-treated groups.

An acute toxicity study was conducted for the assessment of the potential toxicity of TMX, blank albumin-coated SPIONs (BSA-SPIONs), and albumin-coated drug-loaded SPIONs (BSA-SPIONs-TMX) on female Wistar rats. This study involved the investigation of hematological parameters, histopathological evaluation of vital organs, and visual examination. We detected no difference in the behavioral pattern or clinical signs, toxicological effects, or any substantial loss in body weight; a slight weight gain occurred in the treatment group. In all treated groups, no mortality or morbidity was reported during the study period. The four essential organs of the heart, liver, spleen, and kidney were examined histopathologically. As shown in Figure 10, in the TMX-treated group, the section of the heart demonstrated mild disarray, inflammatory infiltrate, and hemorrhage, with a loss of interpolated disc at a few positions, whereas the group treated with BSA-SPIONs showed normal architecture and maintained polarity of myocytes and intercalated disc like the control group. Similarly, in the TMX-treated group, microscopic analysis of the liver showed disorganization in the hepatocytes compared to the control, along with vacuolation in hepatocytes. The liver of the BSA-SPION-treated group revealed an intact liver architecture with a polygonal shape, with round to oval nuclei with coarse chromatin and prominent nucleoli, whereas the group treated with BSA-SPIONs-TMX showed less toxicity in comparison to the TMX-treated group. Normal histology with proper differentiation into red and white cells was observed in a cross-section of the spleen of the control group, and there was no significant change in the histology of the spleen after administering BSA-SPIONs, BSA-SPIONs-TMX, and TMX. Histology of the kidney of the control was normal, and no significant changes in the histology of the kidney were observed in the BSA-SPION group. The BSA-SPION-TMX-treated group showed a normal mesangial matrix, intact vessels, tubules, and glomeruli. In contrast, the TMX-treated group showed slight toxicity, with hemorrhage and degeneration. Overall, BSA-SPIONs-TMX-treated group showed comparatively less toxicity than the group treated with TMX.

## 3. Materials and Methods

### 3.1. Materials

Tamoxifen (purity > 98%) was purchased from Cedilla Pharmaceutical Limited, Ahmedabad, India. Iron (III) chloride hexahydrate (FeCl_3_.6H_2_O) (97%), MTT reagent (2,5-diphenyl tetrazolium bromide) (98%), Rhodamine B (>95%), and sodium hydroxide (NaOH) were obtained from Sigma-Aldrich, Inc., St. Louis, MO, USA. Bovine serum albumin (BSA) was obtained from Santa Cruz Biotechnology Inc. Methanol, and ethanol HPLC grade was procured from SRL (Mumbai, India). The (–) Epigallocatechin-3-gallate (EGCG) (98%) was provided by SSP Private Ltd., New Delhi, India. Analytical grade water was prepared using a Milli-Q Gradient filtration system (Millipore, Burlington, MA, USA). Analytical grade reagents were used throughout the study.

### 3.2. Green Synthesis of Superparamagnetic Iron-Oxide Nanoparticles (SPIONs)

SPIONs were synthesized by the co-precipitation method, involving controlled oxidation reduction of the iron salt precursor using (–) EGCG in aqueous alkaline media at pH 10, maintained by adding 0.5 M NaOH solution as previously described, with slight modifications [67]. Briefly, a solution of EGCG (0.1 M) was added drop by drop to reduce the 0.1 M ferric chloride (III) solution with constant agitation at 500 rpm for 10 min. The color change of the solution from yellow to green to blackish indicated the endpoint of SPION synthesis. The blackish pellets were decanted magnetically and washed using Milli-Q water: ethanol (1:1) to yield the SPIONs after centrifuging at 5000 rpm (Sigma 3k30, Germany) at 4 °C for 10 min. The pellets formed were reconstituted in water for further application.

### 3.3. Preparation of BSA-Coated SPIONs Conjugated with Tamoxifen 

An aqueous solution (10 mL) of the above prepared SPIONs was added dropwise to 10 mL BSA (0.5% *w*/*v*) and TMX (10 mg/mL) solution to obtain BSA-coated SPIONs (BSA-SPIONs-TMX) as previously stated with slight modification [49]. The resultant mixture was agitated at 400 rpm for 30 min, and the pH of the mixture was maintained at pH 10 with 0.5 M NaOH solution, followed by incubation at room temperature for 2–4 h. Then, the BSA-SPIONs were homogenized in a homogenizer to produce uniform SPIONs with the desired particle size before being centrifuged at 30,000 rpm for 1 h at 4 °C under vacuum, using an ultracentrifuge (Optimal LE-80K Beckman Coulter Inc., Brea, CA, USA). The pellets were reconstituted with water, characterized, and freeze-dried using a freeze dryer (VirTis bench top) for storage. 

### 3.4. Characterization of SPIONs and BSA-SPIONs-TMX

The UV-visible absorption spectra of iron-oxide nanoparticles (SPIONs) were determined using a UV-visible spectrophotometer (UV 3600 Shimadzu Little Rock, AR, USA) by scanning the SPIONs between 200 and 600 nm.

The hydrodynamic diameter, polydispersity index (PDI), and zeta potential of BSA-SPIONs-TMX were measured using the zetasizer Nano ZS (Malvern Instruments Ltd., Worcestershire WR14XZ, UK) at 25 °C. All the samples were analyzed for triplicate. Samples were diluted with water at 250× [68]. 

The BSA-SPIONs-TMX were visualized by Transmission Electron Microscopy (TEM) (Technai, G20,20 HR-TEM;(200 Kv) FEI, Hillsboro, OR, USA) at AIIMS, New Delhi, India, after being negatively stained with 2% phosphotungstic acid solution. The surface morphologies of the SPIONs and BSA-SPIONs-TMX were observed by scanning electron microscope (SEM) (ZEISS Gemini 5 1530 FEG, Jena Germany), and quantification of the metal elements in the BSA-SPIONs-TMX was performed using a scanning electron microscope coupled with energy dispersive X-ray (EDX) analysis. 

### 3.5. Estimation of Tamoxifen Content via the HPLC Method

The high-performance liquid chromatography (HPLC) analysis was performed to quantify the tamoxifen content in this study. An HPLC (Shimadzu, Kyoto, Japan) system connected with UV visible light and fitted with a reversed-phase C18 LiChrospher column with a pore size of 5 microns was employed. Mobile phase: water (0.1% triethylamine): methanol (5:95) *v*/*v* with flow rate of 1 mL/min was used, and TMX was quantified at a wavelength of 235 nm. 

### 3.6. Quantification of % Entrapment Efficiency (%EE) 

BSA-SPIONs-TMX (2 mg) were dissolved in 10 mL of methanol and were incubated for 24 h at room temperature using a shaker incubator (Shel Lab, Sheldon, Cornelius, NC, USA). Then, the TMX content was quantified using the above described HPLC method, and the %EE was calculated using the following formula:

(1) $$\%EE=\frac{TMX\;\;in\;the\;BSA-SPIONs-TMX\;}{Total\;TMX\;added\;in\;the\;BSA-SPIONs-TMX}$$

### 3.7. FTIR Spectroscopy

Fourier-transform infrared (FTIR) spectroscopy was performed to confirm the surface coating of SPIONs with BSA, and conjugation of BSA and TMX during the coating process using the KBr pellet technique. For this, the tested powder samples were triturated with KBr in the ratio (1:100 by weight) and pressed into small pellets using a mini-press under extremely high pressure [69]. FTIR spectra were recorded using FTIR (Bruker, Tensor 27, Markham, ON, Canada) at a wavenumber of 400–4000 cm^−1^.

### 3.8. Magnetic Measurement

Magnetic measurements of SPIONs, BSA-SPIONs, and BSA-SPIONs-TMX were evaluated by vibration sample magnetometry (VSM) with the help of lake shore cryotronics, model 7410 series, at a temperature of 300 K. It was performed with a magnetic field of 0–15 KOe, both in increasing and decreasing fields, to the sample kept on a sample holder. The data collected were evaluated with the MicroSense Easy VSM program.

### 3.9. Differential Scanning Calorimetry

Differential scanning calorimetric (DSC) analysis of pure TMX, BSA, and BSA-SPIONs-TMX was performed using a differential scanning calorimeter (Pyris software version 6.0, PerkinElmer, Waltham, MA, USA). The DSC is thermal technique that measures the energy absorbed or emitted by the powder sample as function of temperature. For this, 2–4 mg of the sample was placed into a 40 µL aluminum pan, sealed hermetically, and placed into sample holder of the DSC instrument. During DSC analysis, samples were heated at a rate of 10 °C/min from 30 to 450 °C and supplied with nitrogen at 30 mL/min. A blank aluminum pan was used as the standard control and reference sample. 

### 3.10. X-ray Diffraction

The crystalline patterns of the SPIONs were determined out using an X-ray diffractometer (Ultima IV X-ray diffractometer, Rigaku, Japan) at room temperature with CuK radiation (k = 1.54) operated at 45 kV, 40 mA with a scan speed of 8° min^−1^ in the 2-theta range of 5–60°. A scintillation counter with a K-beta filter was employed as the detector.

### 3.11. Stability Study

The stability study of optimized BSA-SPIONs-TMX was conducted for 90 days, in which lyophilized samples were kept in sealed glass vials and kept at 4 °C and 25 °C/60% RH in a stability chamber (Thermo Lab, Waltham, MA, USA). After different time intervals, each sample was withdrawn, reconstituted, and analyzed for particle size and zeta potential. The samples were reconstituted with Milli-Q water. 

### 3.12. In Vitro Drug Release Study

The in vitro drug release study was performed to determine the release pattern of TMX from the BSA-SPIONs-TMX by using an activated dialysis membrane (MWCO = 12 kDa, Sigma) [70]. The dispersed sample (5 mL) was kept in a dialysis bag and was immersed in 50 mL of phosphate-buffered saline (PBS) pH 7.4 for 24 h at 37 ± 2 °C, with 100 rpm throughout the release study. A total of 500 μL of sample was withdrawn at pre-determined time intervals and replaced with the same amount of fresh PBS solution to maintain the sink conditions, and TMX was quantified using HPLC, as described above.

### 3.13. Intracellular Uptake Study

For the intracellular uptake study, 50 × 10^3^ cells (MCF-7 and T47D) per well were seeded in 12-well plates in DMEM supplemented with 10% FBS and incubated at 37 °C, 5% CO_2_. After 24 h, cells were treated with rhodamine-B-labelled BSA-SPIONs (BSA-SPIONs-Rho-B) and Rho-B plain solution as the control. For rhodamine-B labeling, an aqueous solution (10 mL) of the prepared SPIONs was added dropwise to 10 mL BSA (0.5 % *w*/*v*) and rhodamine-B (1 mg/mL) solution to obtain Rhodamine-labelled BSA-SPIONs. The resultant mixture was incubated in a shaking incubator (Shel Lab, Cornelius, OR, USA) at 100 rpm at room temperature for 2–4 h, centrifuged at 30,000 rpm, and finally pellets were resuspended in water (2.9 mL). After 4 h, cells were washed with sterile PBS, trypsinized, and pelleted via centrifugation, and re-suspended in PBS. Then, the fluorescence intensity was assessed using flow cytometer (Cytoflex, Beckman Coulter, Indianapolis, Indiana, IN, USA), and data were processed via CytExpert software (Indianapolis, Indiana, IN, USA). The M1 event is an indicative value to measure the relative fluorescence by the flow cytometry of the formulation in comparison to rhodamine-B solution. It has no standard value; it is proportional to the fluorescence. For example, if the fluorescence intensity is high than the M1 events are high and vice versa. 

### 3.14. Cell Cytotoxicity Assay

The cell cytotoxicity of the TMX, BSA-SPIONs, and BSA-SPIONs-TMX was assessed using two human estrogen receptor-positive breast cancer cell lines, MCF-7 and T47D. Cells (3 × 10^3^ per well) were cultured in 200 µL of growth medium (DMEM supplemented with 10% FBS) in a 96-well plate and incubated at 37 °C, 5% CO_2_. Then, the cells were treated with different normalized concentrations of TMX (0.1–100 μM) and with blank BSA-SPIONs (equivalent weight to SPIONs) as control.

After 24 h, the media were replaced with a fresh growth medium, and the cells were treated with different concentrations of the samples, with untreated cells used as the control. Then, after 72 h, 20 µL of MTT solution (5 mg/mL) was added to each well and incubated for 3 h at 37 °C before the addition of 150 µL of DMSO to solubilize the formazan crystals. An HT microplate reader was used to measure the absorbance of the sample at 540 nm. The % cell cytotoxicity was calculated using the following formula, and IC_50_ was evaluated using Prism GraphPad.

% Cytotoxicity = [(A − B)/A] × 100(2)

where A and B were the absorbance of untreated and treated cells, respectively.

### 3.15. Acute Toxicity Study

An acute toxicity study was performed on female Wistar rats weighing 150–200 gm, according to the guidelines of Organization for Economic Corporation and Development (OECD). The animals were provided by the central animal house facility, Jamia Hamdard, after the acceptance of the protocol no. 1453 by the IAEC and CPCSEA (Institutional Animal Ethics Committee and Committee for the Purpose of Control and Supervision of Experiments on Animals, respectively). All the animals were kept at standard conditions of temperature 25 ± 2 °C and relative humidity 55 ± 5%. The animals were divided into four groups containing six animals in each group. The rats in group I were taken as the control group and treated by normal saline, group II was given 10 mg/kg tamoxifen suspension, group III was given blank BSA-SPIONs (0.5 mL blank), and group IV was given BSA-SPIONs-TMX (equivalent to 10 mg/kg of TMX). The rats in all the groups were observed twice daily for 14 days for behavioral and clinical signs, toxicological symptoms, and overall appearance, and the weight of the rats was taken prior to treatment, on the 7th day, and on the 14th day [71]. On day 14, the rats were euthanized with ketamine/xylazine, and tissue samples were collected from the heart, liver, spleen, and kidney and kept in 10% formalin solution. The tissue samples were cut into 5 µm thick sections with the help of a rotary microtome (Model MT-1090, Weswox Optik, India) and fixed on glass slides. The glass slides were stained with hematoxylin-eosin (HE) dye and subjected to histopathological evaluation with the help of a microscope. 

### 3.16. Statistical Analysis

The statistical analysis was performed by using GraphPad PrismSoftware (Instat 3.06, San Diego, CA, USA), and a *p* value less than 0.05 was considered statistically significant.

## 4. Conclusions

In the present study, superparamagnetic iron-oxide nanoparticles (SPIONs) were synthesized using green chemistry, with promising properties as a nanocarrier, and were further coated with TMX conjugated to BSA for enhanced stability. The fabricated BSA-SPIONs-TMX exhibited particle size and PDI in the optimum range, and entrapment efficiency was found to be satisfactory as well (Supplementary Materials Figure S1). Additionally, the in vitro drug release studies depicted a sustained release pattern that is of utmost importance for targeted drug delivery systems. Furthermore, the intracellular uptake study conducted on MCF-7 and T47D revealed the higher uptake efficiency of the Rhodamine B-loaded BSA-SPIONs-TMX in contrast to the plain Rhodamine B solution. The MTT assay performed on MCF-7 and T47D further showed the IC_50_ value of 15.12 ± 0.110 μM and 6.29 ± 0.213 μM, respectively, for BSA-SPIONs-TMX, suggesting a higher cell inhibitory effect in terms of cell viability. Moreover, the acute toxicity studies showed the normal histology of different body organs treated by BSA-SPIONs and BSA-SPIONs-TMX in contrast with TMX signifying its safe use as a theranostic tool. However, in vivo studies involving the estimation of various tumor parameters must be performed in future for establishing the efficacy of BSA-SPIONs-TMX over TMX as a treatment option for breast cancer. Furthermore, clinical studies must also be performed for evaluating the effectiveness of the BSA-SPIONs-TMX in humans. 

## Figures and Tables

**Table 1:** Stability study of optimized BSA-SPIONs-TMX at 4 °C and 25 ± 2 °C/60 ± 5% RH for 90 days (*n* = 3) (solvent = Milli-Q water).

Temperature	Time (Days)	Particle Size ± SD nm	Zeta Potential
4 °C	0	116.8 ± 3.8	−30.2 ± 0.1
	45	118.1 ± 5.6	−30.4 ± 0.4
	90	121.9 ± 5.0	−30.6 ± 0.5
25 °C	0	116.8 ± 3.8	−30.2 ± 0.1
	45	121.5 ± 4.8	−30.6 ± 0.04
	90	120.2 ± 5.9	−30.9 ± 0.02

## Data Availability

Not applicable.

## Supplementary Materials

The following supporting information can be downloaded at: https://www.mdpi.com/article/10.3390/molecules28052343/s1, Figure S1: Three-dimensional response surface plots displaying the influence of different process variables on (a) particle size, (b) Zeta potential, and (c) % entrapment efficiency of BSA-SPIONs-TMX.

## Sample Availability

Samples of the compounds are not available from the authors.
