# Supplementary material for: Superparamagnetic Iron-Oxide Nanoparticles Synthesized via Green Chemistry for the Potential Treatment of Breast Cancer
Source: Molecules. 2023 Mar 3;28(5):2343. doi: 10.3390/molecules28052343 (PMC10005561; doi:10.3390/molecules28052343)
Supplement: Supplementary file 1 [file molecules-28-02343-s001.zip › molecules-2163806-supplementary.pdf]

Supplementary data

1. Optimization by Experimental Design

The effect of BSA concentration, homogenization rpm, and time on particle size, PDI, zeta potential, and % EE was determined using design expert software. All other parameters were kept constant throughout the study [25]. The Box Behnken design, BBD is a combination of mathematical and statistical techniques for modelling and examination of issues involving matrices of variables, as well as for other purposes. The quadratic response surface, which was generated using Design expert software, was used to develop the second-order polynomial model (Trial version 13.0.7, Stat-Ease Inc., MN). To determine the main, interactive, and quadratic effect of the formulation variables, the centre points of every corner of the multidimensional cube indicating the area of interest, were employed.

The non-linear quadratic model produced by the design was:

$$H = A_0 + A_1G_1 + A_2G_2 + A_3 G_3 + A_4G_1G_2 + A_5 G_2 G_3 + A_6G_1 G_3 + A_7 G_1^2 + A_8 G_2^2 + A_9 G_3^2 \quad (1)$$

in which H= dependent variables responses; A_0 - A_1 are the regression coefficients of the respective variables; and G_1, G_2, and G_3 represent the coded levels of independent variables [26]. According to Table 1, the factors that are examined in the given study taken as independent variables were BSA concentration (G_1), rpm (G_2), and time (G_3) and were denoted by the numbers 1, 0 and +1 corresponded to the low (G_1), middle (G_2), and high values (G_3), respectively. The dependent variables taken in the study included particle size, PDI, zeta potential, and %EE.

Statistical analysis of the data and validation of the model

The fit of the model was evaluated using design expert software. A comparison of various statistical metrics, including a coefficient of variation (CV), projected r^2, and adjusted r^2 value, was used to select the best-suited model [27]. Multiple regression was used to analyse to generate polynomial models for the response variables, which included quadratic, linear, and interaction factors. The analysis of variance, P, and F values was employed to examine the relevant change in responses due to factors. Contour surface plots depicted the relationship between dependent and independent variables, as well as the effects of multiple factors at the same time.

1.1 Optimization and experimental design

Selected independent variables including BSA concentration, rpm, and time influenced the responses, H_1 (particle size), H_2 (Zeta potential), and H_3 (Entrapment efficiency) as established by the design expert software. The response surface plots were employed to examine the interaction between process variables by keeping one variable constant. The ratios of maximum to minimum responses indicate the need for power transformation. There was no requirement of transformation for particle size, zeta potential, and entrapment efficiency (EE) as the ratio was less than 3. The model source is identified by the sequential model sum of squares, model summary statistics, and lack of fit. The quadratic model was indicated for all the responses as confirmed by the p-value <0.0001, low predicted residual sum of squares (PRESS), and lack of fit. The important factors influencing responses were identified using an analysis of variance. The F-values for various responses H_1, H_2, and H_3 were 10183, 43.52, and 129.06 respectively, confirming the model's significance. The lack of fit was non-significant as implied by the inappropriate F-value. The difference in modified and speculated r^2 values was less than 0.2 for all the responses which indicated the model. The acceptable corrected (signal: noise) values should be greater than 4 for the desired signal. The ratios of 370.05, 23.06, and 32.96 for the three responses indicated an adequate signal [36].

Effects on particle size

The particle size varies from 63.7 to 254.1 nm for various degrees of factor combinations. The particle size of the nanoparticles was affected by independent variables including particle size, the concentration of BSA, time, and rpm, and their effect can be elucidated by the following quadratic equation:

H_1 (Particle size) = $116.84 + 13.16\ G_1 - 25.86G_2 - 17.5G_3 - 0.975\ G_1G_2 - 8.7\ G_1G_3 - 29.05\ G_2G_3 + 78.14G_1^2 - 0.0075G_2^2 + 19.41G_3^2$ (2)

The positive value before the response indicates the positive effect on the factor. The r^2 values was found to be 0.994 which indicated the fit of the model. Figure 1 shows the effect of BSA concentration on particle size by keeping homogenization rpm and time constant. At constant homogenization time and rpm, on increasing the BSA concentration, particle size was decreased initially up to a minimum value and then increased again. Both below and above 0.5% (w/v) BSA concentration, particle size was higher. Capping agents were unable to coat the iron molecules at low BSA concentrations, resulting in aggregation and an increase in particle size, whereas above 0.5 percent extra BSA resulted in accumulation around the iron oxide cores and combining individual SPIONs into an aggregate. The particle size decreased

as the rpm increased at a constant BSA content. Likewise, as the homogenization time increased the particle size decreased. Increased homogenization time and rpm provided energy for the disintegration of aggregates into smaller particles. The homogenization time and rpm affected the particle size of the nanoparticles inversely. Increased PDI was observed with an increase in particle size. The formulation with minimum particle size was selected for optimal formulation characteristics.

Effects on zeta potential (H_2)

The value of zeta potential varies from -25.4 to -40.1 mV under the experimental results. The equation illustrates the effects of several factors on zeta potential:

H_2 (Zeta Potential) = $-30.88-0.4625\ G_1 + 2.40\ G_2 + 1.61\ G_3 - 0.275\ G_1G_2 - 0.75\ G_1G_3 + 2.97\ G_2G_3 - 5.89\ G_1^2 + 0.76G_2^2 - 1.34\ G_3^2$ (3)

The adjusted value was 0.9598 and the predicted r^2 value was 0.8279 indicating that this model fits the study. The effect of different independent variables was determined by keeping one variable constant at a time. The negative coefficient represents an inverse relationship while the positive coefficient depicts a directly proportional effect on the zeta potential. The interaction terms (G_1G_2, G_1G_3, G_2G_3, G_1^2, G_2^2, and G_3^2) demonstrate the change in zeta potential when the two variables are varied simultaneously. The coefficient in the second-order polynomial model showed the effects on zeta potential.

Effects on entrapment efficiency (H_3)

The entrapment efficiency depends on the particle size. The entrapment efficiency ranged from 63.5 to 84.3% for a selected range of variables. The entrapment efficiency depends positively on homogenization rpm and BSA concentration while negatively on homogenization time. The second-order polynomial equation for entrapment efficiency generated by response surface plot with the help of Design expert software is:

H_3 (EE %) = $78.7 + 6.26\ G_1 - 3.76\ G_2 + 2.35\ G_3 - 2.55G_1G_2 + 1.17\ G_1G_3 - 4.03\ G_2G_3 - 3.17\ G_1^2 - 4.98G_2^2 - 1.90\ G_3^2$ (4)

The adjusted was 0.9863 and the predicted r2 value of 0.9222 indicated the model's fit. The coefficients mentioned in the equation second order polynomial demonstrate the effects of the independent variable on entrapment efficiency. The cross-interaction of homogenization rpm and time was significant as the coefficient of G_2G_3 is greater than others. In addition, the coating agent helps to stabilize the nanoparticles preventing their aggregation.

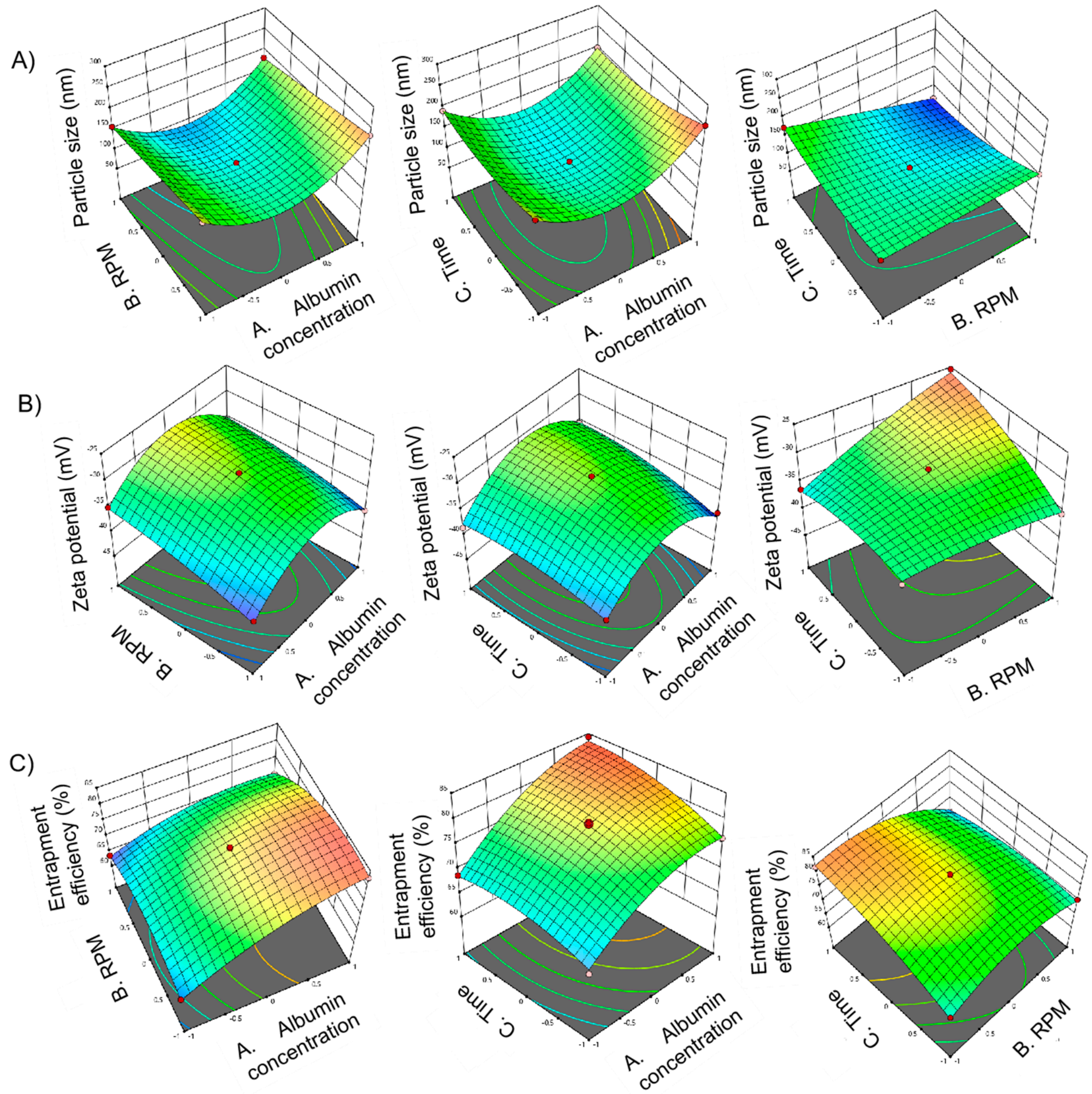

Figure S1: Three-dimensional response surface plots displaying the influence of different process variables on (a) particle size, (b) Zeta potential, and (c) % entrapment efficiency of BSA-SPIONs-TMX
